# Enhancing the therapeutic effects of in vitro targeted radionuclide therapy of 3D multicellular tumor spheroids using the novel stapled MDM2/X-p53 antagonist PM2

**DOI:** 10.1186/s13550-020-0613-7

**Published:** 2020-04-16

**Authors:** Anja C. L. Mortensen, Eric Morin, Christopher J. Brown, David P. Lane, Marika Nestor

**Affiliations:** 1 Department of Immunology, Genetics, and Pathology, The Rudbeck Laboratory, Uppsala University, SE-751 85 Uppsala, Sweden; 2 p53Lab, A*STAR, 8A Biomedical Grove, #06-04/05 Neuros/Immunos, Singapore, 138648 Singapore; 3 Department of Microbiology, Tumor and Cell Biology, Science for Life Laboratory, Karolinska Institutet, Stockholm, Sweden

**Keywords:** PM2, TRNT, Radiosensitization, pErk1/2 induction, 3D tumor models

## Abstract

**Background:**

Precision therapeutics continuously make advances in cancer therapy, and a field of growing interest is the combination of targeted radionuclide therapy (TRNT) with potential radiosensitizing agents. This study evaluated whether the effects of in vitro TRNT, using the ^177^Lu-labeled anti-CD44v6 antibody AbN44v6, were potentiated by the novel stapled MDM2/X-p53 antagonist PM2.

**Materials and methods:**

Two wt p53 cell lines, HCT116 (colorectal carcinoma) and UM-SCC-74B (head and neck squamous cell carcinoma), expressing different levels of the target antigen, CD44v6, were used. Antigen-specific binding of ^177^Lu-AbN44v6 was initially verified in a 2D cell assay, after which the potential effects of unlabeled AbN44v6 on downstream phosphorylation of Erk1/2 were evaluated by western blotting. Further, the therapeutic effects of unlabeled AbN44v6, ^177^Lu-AbN44v6, PM2, or a combination (labeled/unlabeled AbN44v6 +/− PM2) were assessed in 3D multicellular tumor spheroid assays.

**Results:**

Radiolabeled antibody bound specifically to CD44v6 on both cell lines. Unlabeled AbN44v6 binding did not induce downstream phosphorylation of Erk1/2 at any of the concentrations tested, and repeated treatments with the unlabeled antibody did not result in any spheroid growth inhibition. ^177^Lu-AbN44v6 impaired spheroid growth in a dose-dependent and antigen-dependent manner. A single modality treatment with 20 μM of PM2 significantly impaired spheroid growth in both spheroid models. Furthermore, the combination of TRNT and PM2-based therapy proved significantly more potent than either monotherapy. In HCT116 spheroids, this resulted in a two- and threefold spheroid growth rate decrease for the combination of PM2 and 100 kBq ^177^Lu-AbN44v6 compared to monotherapies 14-day post treatment. In UM-SCC-74B spheroids, the combination therapy resulted in a reduction in spheroid size compared to the initial spheroid size 10-day post treatment.

**Conclusion:**

TRNT using ^177^Lu-AbN44v6 proved efficient in stalling spheroid growth in a dose-dependent and antigen-dependent manner, and PM2 treatment demonstrated a growth inhibitory effect as a monotherapy. Moreover, by combining TRNT with PM2-based therapy, therapeutic effects of TRNT were potentiated in a 3D multicellular tumor spheroid model. This proof-of-concept study exemplifies the strength and possibility of combining TRNT targeting CD44v6 with PM2-based therapy.

## Introduction

External beam radiotherapy (EBRT) remains the most commonly used form of radiotherapy for treatment of cancer [1]. However, modern radiotherapy has expanded to more options than EBRT. While radioiodine ([^131^I]-NaI) has been used for the treatment of thyroid cancer for more than half a century, newly approved compounds such as Xofigo® (^223^RaCl_2_) and Lutathera® (^177^Lu-DOTA-Tyr3-Octreotate) have revolutionized the field of radiopharmaceuticals and targeted radionuclide therapy (TRNT) [2–4]. Radioiodine and ^223^RaCl_2_ rely on innate mechanisms to direct their cytotoxic radioactivity to tumor cells. Lutathera® utilizes the presence of specific cell surface antigens predominantly expressed on the tumor cells to deliver the radioactive dose on target, and thereby sparing healthy tissue of unwanted radioactivity. Despite these more recent successes and advances, additional strategies for targeted delivery of radionuclide therapies are needed in order to further extend and tailor the available therapeutic options. Therefore, the search for novel targets and the development of de novo radiopharmaceuticals remain essential.

CD44v6, a splice variant of the CD44s antigen, is a cell surface antigen commonly overexpressed in various cancer types, but it is found only in two subtypes of epithelial cells [5–8]. This pattern of CD44v6 overexpression in cancerous tissues and simultaneously limited expression in adjacent healthy tissues makes it an interesting target for TRNT [9]. In 2003, Börjesson et al evaluated an anti-CD44v6 humanized monoclonal antibody (BIWA 4) labeled with ^186^Re with promising results in patients with head and neck squamous cell carcinoma (HNSCC) [10]. We have separately developed a full-length, fully human recombinant anti-CD44v6 antibody (AbN44v6), which has previously been evaluated for dosimetry and biodistribution in vivo in a small animal tumor model with promising results [11].

While there is no denying that radiotherapy, whether traditional EBRT or by targeted radionuclide therapies, is a successful and effective cancer therapy course, improvements would benefit these therapies. One such improvement is the possibility of combining TRNT with precision drugs that sensitize the cancer cells to the effects of radiation [12]. By sensitizing the tumor prior to the imminent ionizing radiation, radiation doses can be lowered without compromising the level of damage to cancerous tissues. Similarly, potentiating the cytotoxic effects of radiotherapy through radiosensitization, the therapeutic effects of radiotherapy may be improved [13]. Compounds that target the DNA-damage response mechanisms initiated as a result of ionizing radiation are of particular interest for use in combination with radiotherapy [14, 15]. At the very center of the radiation response pathways is the well-known transcription factor p53 [16]. Established as a crucial tumor suppressor, *TP53* is the most commonly mutated gene in all cancers, with a mutation rate of more than 50%. Once mutated, p53 not only loses its ability to suppress tumor growth, but can transform into an oncogene with a plethora of gain-of-function abilities that further enhance tumor growth [17]. In cancers which retain a wild-type p53 (wt p53) expression, the most important negative regulator of p53, mouse-double-minute 2 (MDM2 or HDM2, the human equivalent), is often overexpressed or amplified [18]. MDM2, an E3 ubiquitin ligase, binds to and ubiquitinates p53, inactivating the transcription factor and facilitates its degradation [18, 19]. An overexpression of MDM2 can suppress the otherwise fully functional wt p53 protein, thereby suppressing apoptosis and cell cycle arrest. Several small peptide MDM2-p53 protein-protein interaction antagonists (MDM2-p53 antagonists) are undergoing different stages of clinical trials either as monotherapies or in combination with chemotherapeutic compounds [20]. The concept of inhibiting MDM2 has shown promise so far, albeit not at the expected levels [21]. However, none of the MDM2-p53 antagonists undergoing clinical trials are tested in combination with radiotherapy [20, 21].

PM2 is a novel, stapled peptide that targets the MDM2-p53 protein-protein interaction. Similar to other MDM2-p53 antagonists, PM2 contains three essential amino acids that mimic p53 and bind to the hydrophobic cleft on the MDM2 protein, thus blocking the interaction of MDM2 with its target protein [19]. Contrary to most MDM2-p53 antagonists, PM2 is a dual inhibitor, binding both MDM2 and the structural homologue, MDMX (MDM4) [22]. PM2 was previously evaluated in both an in vitro and in vivo setting of wt p53, HPV-negative cancer cells lines in combination with EBRT with promising results [23, 24].

Combining PM2-based therapy with TRNT is a novel and promising concept. This study assessed the combination of PM2-based therapy and TRNT using AbN44v6 labeled with ^177^Lu in two wt p53, HPV-negative cancer cell lines with moderate and low CD44v6-expression levels using a 3D multicellular tumor spheroid model.

## Materials and methods

### Cell culture

The human colorectal carcinoma HCT116 cell line was purchased from ATCC and cultured in McCoy’s Modified Eagle Medium with 10% fetal bovine serum (FBS), 1% l-Glutamine and 1% antibiotics (100 IU penicillin and 100 μg/ml streptomycin). The human squamous cell carcinoma cell line UM-SCC-74B, kindly provided by Professor TE Carey (University of Michigan, MI, USA), was cultured in Dulbecco’s Modified Eagle Medium (DMEM) with 10% FBS, 1% l-Glutamine, 1% antibiotics (100 IU penicillin and 100 μg/ml streptomycin) as well as 1% non-essential amino acids. Starvation medium contained the above additives with the exception of FBS. Previous studies by our group have shown that HCT116 can be considered a moderate CD44v6-expressing cell line and UM-SCC-74B a low CD44v6-expressing cell line [11]. Cells were incubated at 37 °C with 5% CO_2_ and cultured for no longer than 3 months.

### Antibodies and PM2

AbN44v6, a fully human recombinant, full-length antibody targeting CD44v6, was developed from the CD44v6-targeting Fab-fragment AbD15179 and has previously been described [11, 25]. It was supplied in borate buffer at 3 mg/ml by Bio-Rad AbD Serotec (Puchheim, Germany). The commercially available anti-CD20 antibody, rituximab (MabThera), was purchased from Apoteket AB (Stockholm, Sweden) and used as a negative control. U36 was kindly provided by Professor G.A.M.S. van Dongen (VU University Medical Center, Amsterdam, the Netherlands). For Western blotting, primary antibodies, rabbit anti-phosphoErk and rabbit anti-Erk (Cell Signaling Technology, MA, USA), and mouse anti-β-catenin (BD biosciences, CA, USA) as well as secondary antibodies horse-radish peroxidase-conjugated secondary antibodies (GE Healthcare, UK) were used. The novel, stapled MDM2/X-p53 antagonist, PM2 (Mw = 1462.75 Da), was produced at the p53 Laboratory (A*STAR, Singapore) and dissolved in DMSO to a stock concentration of 10 mM and stored at − 20 °C.

### Western blot

Approximately 7.5 × 10^4^ HCT116 or 5 × 10^4^ UM-SCC-74B cells were seeded in 6-well plates and incubated at 37 °C with 5% CO_2_ to a confluency of 50–60% before switching to starvation medium. Cells starved overnight were treated or not with fibroblast growth factor 2 (FGF2), 10 ng/ml for 5 min, and antibodies, as indicated, thereafter lysed in Nonidet P-40 (NP-40) lysis buffer: 50 mM HEPES pH 7.5, 100 mM NaCl, 1 mM EGTA, 1 mM PMSF, 5 μg/ml aprotinin, 5 μg/ml leupeptin, 100 μM Na_3_VO_4_, and 1% NP-40. Lysates were subjected to SDSPAGE (Invitrogen by Thermo Fisher, CA, USA), followed by transfer to Hybond-C extra membranes (Amersham Biosciences, Uppsala, Sweden). Membranes were incubated with indicated primary antibodies followed by horse-radish peroxidase-conjugated secondary antibodies. Immune reactivity was visualized using the enhanced chemiluminescence plus detection system (GE healthcare, UK). Quantification of immunoblotting signals was done using the Image Lab software (Bio-Rad Laboratories, CA, USA).

### Radiolabeling and EDTA challenge

^177^Lu was purchased from PerkinElmer (Waltham, MA, USA) as LuCl_3_. AbN44v6 (4 mg/ml) and rituximab (5 mg/ml) were chelated for 4 h at 37 °C in 0.07 M sodium borate, pH 9.2, with CHX-A”DTPA containing a molar ratio of 1:5 between antibody and CHX-A”DTPA. Prior to radiolabeling, excess CHX-A”DTPA was separated from the antibodies using NAP-5 size exclusion chromatography columns (GE Healthcare, Uppsala, Sweden) equilibrated with filtered, metal-free 0.2 M ammonium acetate (pH 5.5, stored over Chelex 100). For AbN44v6, 11-30 MBq of ^177^LuCl_3_ was added to between 200 and 375 μg of AbN44v6 chelated with CHX-A”DTPA and incubated for 1 h at room temperature. For Rituximab, 5-40 MBq of ^177^LuCl_3_ was added to 100-200 μg of antibody and incubated for 1 h at room temperature. Labeled antibodies were separated from radionuclide and low-molecular-weight reaction components by using a NAP-5 column equilibrated with PBS. To determine the yield, purity, and stability of the final product, Instant Thin Layer Chromatography (ITLC) analyses were performed. Approximately 0.5 μl of the mixture was placed on a chromatography strip (Biodex, Shirley, NY, USA), using 0.2 M citric acid as the mobile phase, followed by measurements on a Cyclone Storage Phosphor System (PerkinElmer). The data were analyzed using the OptiQuant image analysis software (PerkinElmer). Yields were defined as fraction (percentage) of radiolabeled antibody versus unlabeled, free ^177^Lu. NAP-5 purification resulted in > 95% purity for all samples post labeling. For 3D cell culture assays, the amount of free ^177^Lu was adjusted for all samples in order to ensure equal experimental conditions.

An ethylenediaminetetraacetic acid (EDTA) challenge assessed the stability of the ^177^Lu-labeled antibody by ITLC, using a 500:1-M ratio with antibody and incubated at 37 °C for up to 48 h.

### Specificity assay

Approximately 3 × 10^4^ HCT116 or UM-SCC-74B cells per well were seeded in 48-well plates and incubated for 24 h at 37 °C with 5% CO_2_ before 20 kBq of ^177^Lu-AbN44v6 (27 nM) was added to each well. Additionally, 100-fold excess of unlabeled AbN44v6 was added to selected wells in order to block specific binding. The 48-well plates were incubated at 37 °C with 5% CO_2_ for 24 h and then washed, harvested, and measured in a 1480 WIZARD gamma well-counter (Wallac Oy, Turku, Finland).

### LigandTracer analyses

All LigandTracer experiments were performed on LigandTracer Yellow (Ridgeview Instruments, Vänge, Sweden). Approximately 5 × 10^5^ HCT116 or UM-SCC-74B cells were seeded on tilted petridishes 24 h prior to the start of each assay and incubated at 37 °C with 5% CO_2_. ^177^Lu-AbN44v6 was added in two concentrations (10 and 30 nM), and the uptake was measured at room temperature for 90 min for each concentration followed by a dissociation phase of at least 10 h. Analysis was performed using TraceDrawer version 1.7 (Ridgeview Instruments, Vänge, Sweden).

### 3D cell culture assay

For liquid overlay of two flat-bottomed 96-well plates, 0.15 g of agarose was dissolved in 10 ml of PBS, pH 7.4, with 1% penicillin/streptomycin and 5% incomplete, serum-free DMEM. Fifty microliters of the solution was added to each well prior to seeding 10^3^ HCT116 or UM-SCC-74B cells. The plates were incubated at 37 °C with 5% CO_2_ for at least 3 days prior to start of treatment with ^177^Lu-labeled antibodies and/or PM2. The 3-day incubation allowed spheroids to form and grow to a preferred size with a diameter of approximately 400 μm. 30 kBq or 100 kBq of ^177^Lu-labeled antibodies were added per well (40–140 nM and 27–79 nM of AbN44v6 and rituximab, respectively), whereas a previously determined, fixed concentration of PM2 (20 μM) was used for all drug treated wells. For treatment with unlabeled AbN44v6, 10 nM, 100 nM, or 1 μM of AbN44v6 was added thrice at 48 h intervals. Spheroid images were obtained at start of treatment and at 2-to-4-day intervals during growth monitoring using a Canon EOS 700D digital camera mounted on an inverted Nikon Diaphot-TMD microscope. Spheroid volumes were calculated by measuring the surface areas using the ImageJ software, version 1.48 (NIH, Bethesda, MD, USA), and assuming all spheroids retained a spherical form, calculated the volumes accordingly using the formula: $V=\frac{4}{3}\pi {r}^3$. For each time point, the growth ratio was calculated by normalizing the volume of the spheroid to the original volume at the start of treatment.

### Statistical analyses

For statistical analyses, one-way ANOVA followed by Tukey’s multiple comparisons test using the Graphpad Prism software version 6.0 (San Diego, CA, USA) was used for comparisons between multiple groups. Student’s *t* test was used for comparisons between two groups. A *p* value < 0.05 was considered significant.

## Results

### Radiolabeling

Average labeling yields of ^177^Lu-AbN44v6 were 71% ± 9%, with an average specific activity of 66 kBq per μg AbN44v6. Average labeling yields for ^177^Lu-rituximab were 73% ± 11%, with an average specific activity of 83 kBq per μg. EDTA challenges resulted in 100% stability at 48 h post labeling.

### AbN44v6 specificity and antigen density

**Fig. 1:**
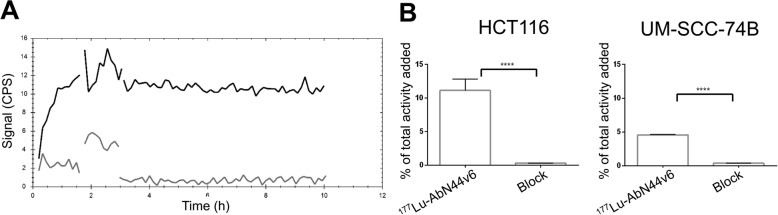
**a** LigandTracer curve representing ^177^Lu-AbN44v6 uptake and retention of HCT116 (black) and UM-SCC-74B (gray). **b** Specificity evaluation of ^177^Lu-AbN44v6 using HCT116 and UM-SCC-74B. *p* < 0.0001 (****), *n* ≥ 3, error bars presented as SD

LigandTracer experiments demonstrated a clear binding of ^177^Lu-AbN44v6 (10 nM and 30 nM) to HCT116 cells followed by a slow dissociation phase. The signal of bound ^177^Lu-AbN44v6 to UM-SCC-74B cells was below the detection limit of the instrument, likely due to the low amount of target antigens expressed on the cell line (Fig. 1a). Specificity assays demonstrated the presence of CD44v6-receptors on both cell lines, since uptake could be blocked by 100-fold excess of unlabeled AbN44v6 (*p* < 0.0001, Fig. 1b). The higher uptake on HCT116 cells was in line with the LigandTracer results, further indicating the presence of a greater abundance of CD44v6-receptors on the HCT116 cells compared to UM-SCC-74B cells in a monolayer setting.

### Effects of unlabeled AbN44v6 and PM2-monotherapy

**Fig. 2:**
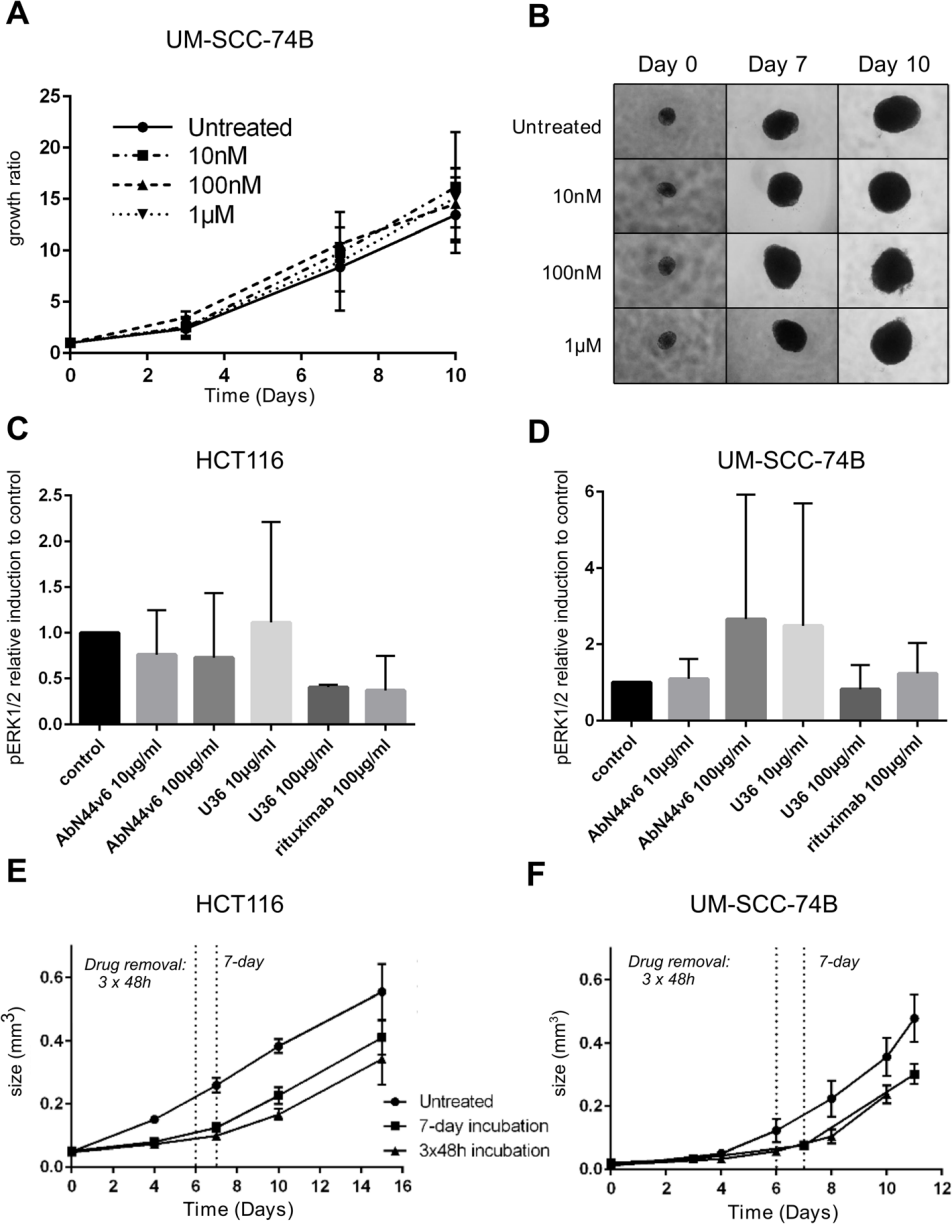
**a** Dose-response of AbN44v6 on UM-SCC-74B spheroids treated with AbN44v6 (untreated controls, 10 nM, 100 nM, 1 μM) presented as growth ratios over time as well as **b** representative images of treated spheroids, *n* ≥ 4, error bars presented as 95% confidence intervals. **c** Relative pErk1/2 induction following FGF2 stimulation compared to control of HCT116 samples. **d** Relative pErk1/2 induction following FGF2 stimulation compared to control of UM-SCC-74B samples, *n* = 3, error bars presented as SD. **e** Growth inhibitory effects of PM2 measured in spheroid volume (mm^3^) using two different treatment regimens on HCT116 spheroids and **f** UM-SCC-74B spheroids. *n* ≥ 5, error bars presented as 95% confidence intervals

AbN44v6-therapy (unlabeled antibody) did not influence the growth of UM-SCC-74B spheroids at any of the added concentrations (Fig. 2a, b). Western blot following FGF2-stimulation (Fig. 2c, d) demonstrated that AbN44v6 did not affect downstream signaling in the Erk1/2 pathway as the relative induction of pErk1/2 was similar when comparing AbN44v6 with U36 (anti-CD44v6 antibody) or negative control (anti-CD20 antibody, rituximab) in either cell line. Neither differences in the absolute (data not shown) nor relative induction of pErk1/2 differed significantly between controls and AbN44v6-treated samples in either cell line.

Incubation with a single dose of PM2 (20 μM) for seven days significantly impaired spheroid growth of both cell lines (Fig. 2e, f). Interestingly, three repeated doses of PM2 (20 μM) at 48-h intervals demonstrated similar growth inhibitory effects as a single dose incubated for seven days (Fig. 2e, f). By day 10, spheroid growth rates were significantly impaired and the growth was continuously affected throughout the end of the assay for UM-SCC-74B spheroids, whereas the HCT116 spheroids regained normal growth rates following removal of PM2.

### Therapy of 3D multicellular tumor spheroids

#### TRNT and combination theapy of HCT116 spheroid

**Fig. 3:**
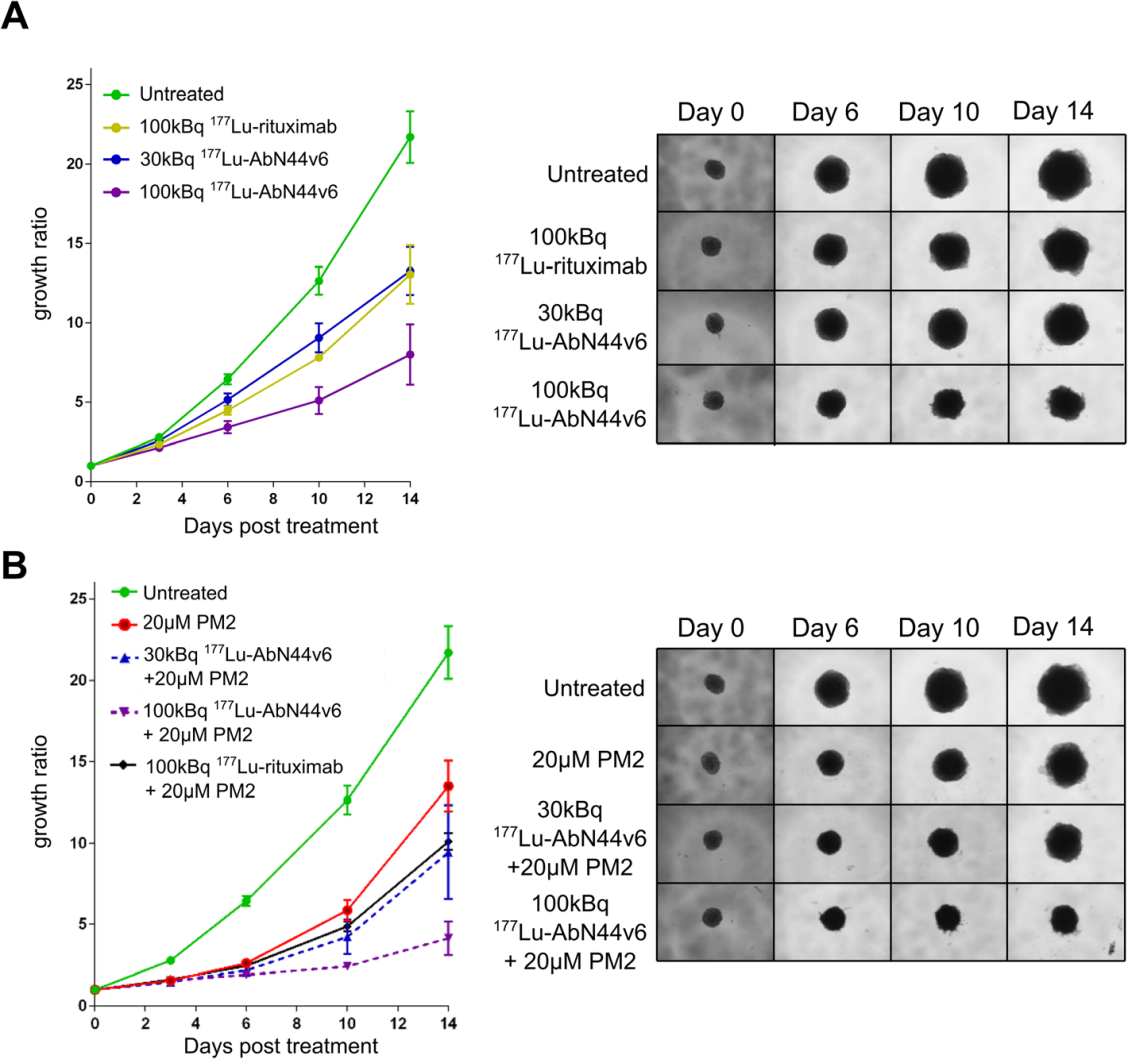
**a** HCT116 spheroid growth over time after treatment with 30 kBq and 100 kBq ^177^Lu-AbN44v6 and 100 kBq ^177^Lu-rituximab as well as representative images of treated spheroids. **b** HCT116 spheroid growth over time after treatment with 20 μM of PM2 and the combination of 20 μM PM2 with 30 kBq and 100 kBq ^177^Lu-AbN44v6 and 100 kBq ^177^Lu-rituximab as well as representative images of treated spheroids. *n* ≥ 4, error bars presented as 95% confidence intervals. A combination of 20 μM PM2 with 30 kBq ^177^Lu-AbN44v6 differed significantly from monotherapies (*p* < 0.01 and *p* < 0.05 for PM2 and TRNT, respectively). A combination of 20 μM PM2 with 100 kBq ^177^Lu-AbN44v6 differed significantly from monotherapies (*p* < 0.0001 and *p* < 0.05 for PM2 and TRNT, respectively) as well as from the combination of 20 μM PM2 with ^177^Lu-rituximab (*p* < 0.0001), as assessed with One-way ANOVA followed by Tukey’s multiple comparisons test

The growth rates of HCT116 spheroids were affected by ^177^Lu-AbN44v6 in an activity-dependent manner (Fig. 3a, Table 1). At the end of the assay (day 14), treatment with ^177^Lu-AbN44v6 resulted in a 40% (30 kBq) and 60% (100 kBq) decrease in spheroid growth compared to untreated controls (*p* < 0.0001). Since the presence of ^177^Lu in the cell medium is expected to contribute to the total radiation dose received by the spheroids, the negative control ^177^Lu-rituximab was also expected to affect spheroid growth to some extent. By day 14, treatment with 100 kBq ^177^Lu-rituximab resulted in a 40% decrease in spheroid growth compared to untreated controls (*p* < 0.0001). Thus, ^177^Lu-rituximab-treated spheroids were significantly larger than spheroids treated with the same dose of ^177^Lu-AbN44v6 (*p* < 0.001, Fig. 3a, Table 1). Furthermore, a single dose of PM2 (20 μM) impaired HCT116 spheroid growth (*p* < 0.0001 Fig. 3b, Table 1), with mean spheroid growth ratios approximately 40% lower than untreated controls at the endpoint. The combination of PM2 and ^177^Lu-AbN44v6 resulted in significantly greater inhibitory effects on spheroid growth rates compared to either monotherapy (*p* < 0.05, Fig. 3b, Table 1). For the combination of 100 kBq ^177^Lu-AbN44v6 and PM2, spheroid growth was significantly decreased compared to untreated controls as well as to PM2 and ^177^Lu-AbN44v6-monotherapies. Additionally, the combination of PM2 and 100 kBq ^177^Lu-rituximab resulted in significantly slower growth rates than untreated controls, although no significance was obtained when compared to monotherapies.

**Table 1:** HCT116 spheroid growth over time in mean and SD. *n* ≥ 4

	Untreated		100 kBq ^177^Lu-rituximab		PM2 [20 μM]		30 kBq ^177^Lu-AbN44v6		100 kBq ^177^Lu-AbN44v6		30 kBq ^177^Lu- AbN44v6 and PM2		100 kBq ^177^Lu-AbN44v6 and PM2	
Day	Mean	SD	Mean	SD	Mean	SD	Mean	SD	Mean	SD	Mean	SD	Mean	SD
3	2.79	0.14	2.34	0.1	1.56	0.11	2.56	0.13	2.14	0.16	1.47	0.04	1.56	0.01
6	6.44	0.43	4.47	0.21	2.61	0.19	5.15	0.32	3.42	0.32	2.18	0.22	1.92	0.15
10	12.64	1.14	7.81	0.09	5.87	0.74	9.06	0.75	5.1	0.69	4.23	0.68	2.43	0.16
14	21.72	2.17	13.04	1.48	13.5	1.88	13.27	1.22	8	1.53	9.43	1.82	4.15	0.66

#### TRNT and combination therapy of UM-SCC-74B spheroids

**Fig. 4:**
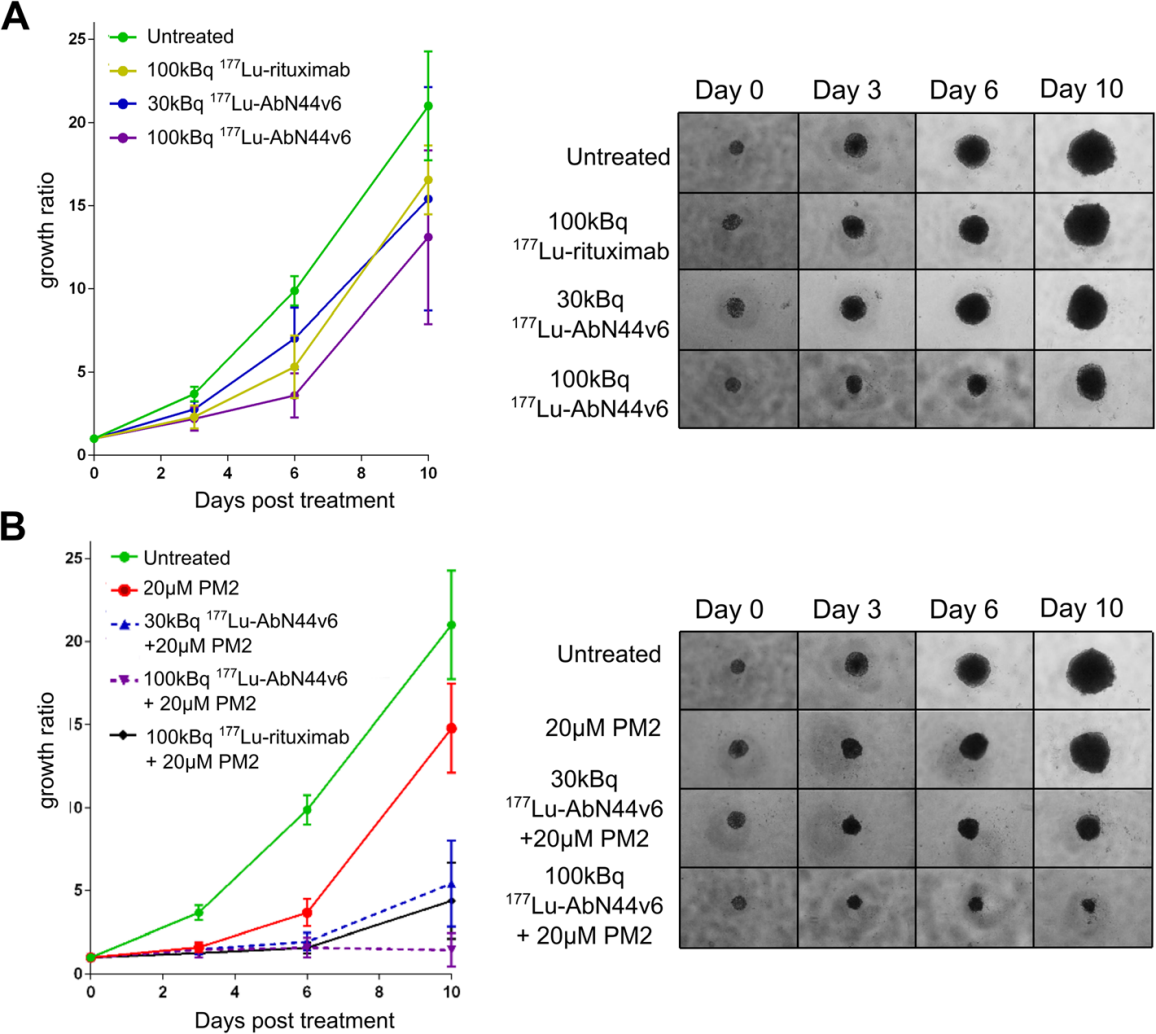
**a** UM-SCC-74B spheroid growth over time after treatment with 30 kBq and 100 kBq ^177^Lu-AbN44v6 as well and 100 kBq ^177^Lu-rituximab as well as representative images of treated spheroids. **b** UM-SCC-74B spheroid growth over time after treatment﻿ with 20 μM of PM2 and the combination of 20 μM PM2 with 30 kBq and 100 kBq ^177^Lu-AbN44v6 and 100 kBq ^177^Lu-rituximab as well as representative images of treated spheroids. *n* ≥ 4, error bars presented as 95% confidence intervals. A combination of 20 μM PM2 with 30 kBq ^177^Lu-AbN44v6 differed significantly from monotherapies (*p* < 0.001). A combination of 20 μM PM2 with 100 kBq ^177^Lu-AbN44v6 differed significantly from monotherapies (*p* < 0.0001 and p < 0.001 for PM2 and TRNT, respectively). The combination of 20 μM PM2 with 100 kBq ^177^Lu-rituximab did not differ from combination therapy of PM2 with ^177^Lu-AbN44v6 as assessed with one-way ANOVA followed by Tukey’s multiple comparisons test

CD44v6-targeted TNRT did not affect the UM-SCC-74B spheroids to the same extent as the HCT116 spheroids. Treatment with 30 kBq ^177^Lu-AbN44v6 failed to significantly impair UM-SCC-74B spheroid growth. The higher dose of ^177^Lu-AbN44v6 (100 kBq) resulted in a decrease in mean spheroid size by approximately 40% of untreated controls (*p* < 0.01, Fig. 4b, Table 2). However, the decrease in size did not differ significantly from non-specific TRNT using 100 kBq ^177^Lu-rituximab (Fig. 4a, Table 2). PM2-based therapy affected UM-SCC-74B spheroids in a similar manner as the HCT116 spheroids, with a mean spheroid size approximately 30% smaller than untreated controls at the endpoint (Fig. 4b, Table 2). While targeted TRNT only resulted in modest effects on UM-SCC-74B, a clear activity-dependent growth inhibition was observed once PM2 was combined with TRNT. The spheroid growth ratio of 30 kBq ^177^Lu-AbN44v6 was decreased threefold upon the addition of a single dose of PM2 (20 μM) compared to TRNT alone (*p* < 0.001 mean size 26% ± 13% of untreated controls). Furthermore, the combination treatment of 100 kBq ^177^Lu-AbN44v6 and PM2 decreased spheroid growth sizes tenfold compared to monotherapies (*p* < 0.001), demonstrating completely stalled spheroid growth and signs of disintegration (Fig. 4b, Table 2).

**Table 2:** UM-SCC-74B spheroid growth ratio-development over time in mean and SD. *n* ≥ 4

	Untreated		100 kBq ^177^Lu-rituximab		PM2 [20 μM]		30 kBq ^177^Lu-AbN44v6		100 kBq ^177^Lu-AbN44v6		30 kBq ^177^Lu-AbN44v6 & PM2		100 kBq ^177^Lu-AbN44v6 & PM2	
Day	Mean	SD	Mean	SD	Mean	SD	Mean	SD	Mean	SD	Mean	SD	Mean	SD
3	3.81	0.37	2.30	0.57	1.53	0.23	2.73	0.43	2.19	0.57	1.48	0.25	1.45	0.30
6	9.87	0.71	5.32	1.52	3.4	0.43	6.69	1.49	3.6	1.07	1.95	0.43	1.59	0.38
10	21.01	2.63	16.57	1.67	14.98	2.15	15.4	2.79	13.12	4.21	5.43	2.09	1.44	0.63

## Discussion

While PM2-based therapy has previously been examined in combination with EBRT both in vitro and in vivo [23, 24], combining the peptide with TRNT is unchartered territory. One important aspect for feasible TRNT is a suitable target antigen, preferably expressed exclusively on the cancerous tissue. CD44v6, a cancer-associated antigen with limited expression in healthy tissues, has previously been shown to be a suitable antigen for this purpose [5–8, 26].

In the present study, the low CD44v6-expressing cell line UM-SCC-74B and moderate CD44v6-expressing cell line HCT116 were used as model systems. The cell lines were chosen based on their wt p53-status, CD44v6 expression, and suitability for use in 3D models. Furthermore, as TRNT is primarily assessed in high antigen-expressing cell lines, investigating whether TRNT is applicable in a moderate antigen-expressing cell line was of interest. Antigen density and antibody specificity were first verified using specificity assays and LigandTracer analyses (Fig. 1a, b). Specificity assays demonstrated that ^177^Lu-AbN44v6 bound specifically to CD44v6, and that CD44v6-antigens were present on both cell lines, albeit in a greater amount on the HCT116 cells. The higher CD44v6-density on HCT116 cells was in line with results from LigandTracer analyses, where a clear signal was detected from HCT116 cells, whereas the signal from UM-SCC-74B cells was below the detection limit of the LigandTracer instrument. This supports previous studies assessing the CD44v6-antigen expression of these cell lines, verifying that CD44v6-antigen density on HCT116 and UM-SCC-74B cells can be considered moderate and low, respectively [27–29].

The effects of cold (i.e., non-radiolabeled) AbN44v6 have not previously been examined. CD44v6 is reportedly involved in invasion and metastasis, and antibody binding could potentially influence the proliferative rate of CD44v6-positive cancer cells [30]. Repeated treatments with cold AbN44v6 did not affect the growth of UM-SCC-74B spheroids in neither an inhibitory nor a proliferative manner (Fig. 2a, b). Additionally, treatment with excessive concentrations of AbN44v6 (100 μg/ml) did not affect phosphorylation of Erk1/2 following FGF2 stimulation on either cell line (Fig. 2c, d). This is in contrast to a study by Orian-Rousseau et al. 2002, where CD44v6-stimulation in combination with hepatocyte growth factor resulted in increased pErk1/2 signaling of the HT29 cell line [31]. The relative induction of pErk1/2 upon FGF2 stimulation seen in Fig. 2c, d following AbN44v6-treatment did not differ from that of negative control (rituximab) nor U36 (positive control), suggesting no direct effect on downstream signaling. The lack of downstream signaling indicates that AbN44v6 could be utilized without inducing proliferating effects.

Monotherapy using PM2 resulted in a clear inhibitory effect on the growth of both HCT116 and UM-SCC-74B spheroids. Interestingly, a single dose with a prolonged incubation time (7 days) demonstrated a similar impact on growth as three repeated doses at 48-h intervals on both HCT116 (Fig. 2e) and UM-SCC-74B (Fig. 2f) spheroids. The similar impacts on growth of a single dose and a thrice-repeated dose on both cell lines points to an extended biological half-life of PM2 compared to other MDM2-p53 antagonists [19].

As HCT116 cells express more CD44v6-antigens than UM-SCC-74B cells, it was expected that the former would be more severely affected by TRNT using AbN44v6. As hypothesized, a large part of the growth inhibitory effects observed on the HCT116 spheroids was due to targeted, as opposed to non-specific, radiation (Fig. 3a, Table 1). More than three times the activity (100 kBq) of non-specific radioactivity (i.e., ^177^Lu-rituximab) was needed in order to obtain a similar impact as ^177^Lu-AbN44v6 (30 kBq). In contrast, the majority of the growth inhibition attained on the low CD44v6-expressing UM-SCC-74B spheroids was likely caused by the mere presence of radiation in the cell medium. As a result, no significant differences were detected between targeted and non-specific radiation on the UM-SCC-74B spheroids (Fig. 4a). This confirms that the targeted effect of the TRNT is linked to the CD44v6 density of the cancer cells. These findings were consistent with previous studies using AbN44v6 for in vitro TRNT in spheroid models, where HCT116 responded in a superior manner to AbN44v6 TRNT compared to UM-SCC-74B [11]. The effect of PM2-based monotherapy was similar between the two cell lines (Figs. 3b and 4b, Tables 1 and 2). The mean growth ratio of PM2-treated samples at the endpoint was approximately 64% and 71% of untreated controls for HCT116 and UM-SCC-74B spheroids, respectively (Tables 1 and 2). The greatest impact on spheroid growth, however, was observed in the combination treated samples (Figs. 3b and 4b). PM2-based therapy strongly potentiated the effects of TRNT on the HCT116 spheroids. Combination-treated HCT116 spheroids were significantly smaller at nearly all time points assessed than spheroids treated with either TRNT or PM2. These results indicate that TRNT in combination with a radiosensitizing agent such as PM2 may be a feasible future strategy for CD44v6-expressing cancers.

The addition of a single dose of PM2 to UM-SCC-74B spheroids resulted in a distinct radiosensitizing effect, though the radiation dose was primarily non-specific. The size of UM-SCC-74B spheroids treated with 100 kBq ^177^Lu-AbN44v6 combined with PM2 were reduced over time and started to disintegrate completely at the endpoint. UM-SCC-74B cells have previously been shown to express high levels of Bcl-2 [32, 33]. This renders enhanced MDM2 expression levels via the activation of the Raf-MEK-ERK pathway, independent of the otherwise crucial p53 activation and accumulation [32, 33]. Consequently, in the presence of overexpressed MDM2 protein levels, governed by sequestered factors, any activation of p53 will be inhibited and ubiquitinated. Thus, we hypothesize that the sensitivity of UM-SCC-74B spheroids to PM2-based therapy is likely linked to the innate Bcl-2 expression and subsequent MDM2 overexpression. Thus, by utilizing PM2 to inhibit MDM2, as well as inducing a p53 response through irradiation, the growth of UM-SCC-74B spheroids could be markedly affected by the combination therapy. The observed pronounced sensitivity of UM-SCC-74B to the combination of radiation and PM2-based therapy is in line with previous research by our group when combining external radiation with PM2 [23, 24]. These data illustrate the potency and potential of MDM2-inhibition, particularly in combination with radiotherapy. However, regardless of the impressive effects of the combination therapy on UM-SCC-74B in vitro demonstrated in the present study, it would translate poorly into an in vivo setting with a high risk of off-target side effects, since the radiation dose to these spheroids was primarily non-specific. This suggests that wt p53 cells expressing low amounts of targeted antigens will primarily benefit from a combination of PM2 and external radiation, whereas for wt p53 cells expressing high levels of the targeted antigen, a combination of PM2 with TRNT remains plausible and warrants further investigation in an in vivo setting.

## Conclusion

In conclusion, the present study assessed the potential of combining CD44v6-targeted TRNT with PM2-therapy in wt p53 cancers, using a 3D multicellular tumor spheroid model. Whereas monotherapies demonstrated dose dependent growth inhibitory effects, the most pronounced effects were obtained in the combination treated groups. This proof-of-concept study exemplifies the potential of combining TRNT with PM2-based therapy. However, further in vivo studies are warranted to confirm these promising findings.

## Data Availability

All data and materials are included in the manuscript unless otherwise stated.

## Abbreviations

EBRT: External beam radiotherapy
FGF2: Fibroblast growth factor 2
HPV: Human papilloma virus
MDM2: Mouse-double-minute 2
TRNT: Targeted radionuclide therapy
